# Orally Active Multi-Functional Antioxidants Are Neuroprotective in a Rat Model of Light-Induced Retinal Damage

**DOI:** 10.1371/journal.pone.0021926

**Published:** 2011-07-14

**Authors:** James Randazzo, Zifeng Zhang, Michael Hoff, Hiroyoshi Kawada, Andrew Sachs, Yang Yuan, Neena Haider, Peter Kador

**Affiliations:** 1 Department of Pharmaceutical Sciences, University of Nebraska Medical Center, Omaha, Nebraska, United States of America; 2 Department of Genetics, Cell Biology and Anatomy, University of Nebraska Medical Center, Omaha, Nebraska, United States of America; 3 Department of Ophthalmology, University of Nebraska Medical Center, Omaha, Nebraska, United States of America; Institute Biomedical Research August Pi Sunyer (IDIBAPS) - Hospital Clinic of Barcelona, Spain

## Abstract

**Background:**

Progression of age-related macular degeneration has been linked to iron dysregulation and oxidative stress that induce apoptosis of neural retinal cells. Since both antioxidants and chelating agents have been reported to reduce the progression of retinal lesions associated with AMD in experimental animals, the present study evaluates the ability of multi-functional antioxidants containing functional groups that can independently chelate redox metals and quench free radicals to protect the retina against light-induced retinal degeneration, a rat model of dry atrophic AMD.

**Methods/Results:**

Proof of concept studies were conducted to evaluate the ability of 4-(5-hydroxypyrimidin-2-yl)-N,N-dimethyl-3,5-dioxopiperazine-1-sulfonamide (compound **4**) and 4-(5-hydroxy-4,6-dimethoxypyrimidin-2-yl)-N,N-dimethyl-3,5-dioxopiperazine-1-sulfonamide (compound **8**) to reduce retinal damage in 2-week dark adapted Wistar rats exposed to 1000 lx of light for 3 hours. Assessment of the oxidative stress markers 4- hydroxynonenal and nitrotyrosine modified proteins and Thioredoxin by ELISA and Western blots indicated that these compounds reduced the oxidative insult caused by light exposure. The beneficial antioxidant effects of these compounds in providing significant functional and structural protection were confirmed by electroretinography and quantitative histology of the retina.

**Conclusions/Significance:**

The present study suggests that multi-functional compounds may be effective candidates for preventive therapy of AMD.

## Introduction

Age-related macular degeneration (AMD), the leading cause of blindness in people over the age of 65 in the United States [1], [2], occurs in two major forms. The first is a geographic atrophy (‘dry’) form that is defined by degeneration of photoreceptors and the retinal pigmented epithelium (RPE) near the macula, the accumulation of lipofuscin (A2E), and the formation of drusen. The second is a ‘wet’ form that is associated with choroidal neovascularization (CNV) [3]. While a number of anti-neovascular agents are clinically used to treat the CNV associated with the wet form of AMD, there are no clinically approved agents to treat geographic atrophy, which accounts for 45% of all advanced AMD cases [4].

Animal models currently used to identify and evaluate new therapies for AMD include transgenic, immunized, and natural murine models; laser photocoagulation and natural primate models; and laser-induced neovascularization and light-induced retinal damage rat models [3], [5], [6]. In the rat, light-induced retinal degeneration mimics changes associated with late-stage, atrophic retinal degeneration that include RPE ablation and photoreceptor cell death [6]. Although the exact mechanism(s) of light-induced photoreceptor damage have not been established, continuous visible irradiation has been suggested to generate superoxide and promote iron release from ferritin [7], [8], [9]. Together, the free iron and superoxide anions can undergo the Haber-Weiss and Fenton reactions to generate damaging hydroxyl radicals [10], that lead to lipid peroxidation of retinal tissues [11], [12], [13] and plasma membrane injury [12], [14], [15], [16]. To prevent photoreceptor apoptosis associated with oxidative stress, both iron chelators, such as desferrioxamine [17] that reduce the levels of free iron, and a wide range of natural and synthetic antioxidants [18], [19], [20], [21], [22] have been investigated.

Recently, we have reported the synthesis of multi-functional antioxidants, 4-(5-hydroxypyrimidin-2-yl)-N,N-dimethyl-3,5-dioxopiperazine-1-sulfonamide and 4-(5-hydroxy-4,6-dimethoxypyrimidin-2-yl)-N,N-dimethyl-3,5-dioxopiperazine-1-sulfonamide, labeled compounds 4 and 8, respectively [23] (Table 1). These compounds contain both a unique 2-amino-5-hydroxy-1,3-pyrimidine free radical scavenger and the independent ability to selectively chelate metal ions. Together, they reduced reduce Fenton-generated reactive oxygen species (ROS)-induced RPE damage in vitro [23]. Here, we report the first in vivo evaluation of oral administration of compounds possessing this unique scavenging system with the independent ability to attenuate redox-active metals in a light-induced retinal damage rat model. In this study, retinal neuroprotection was assessed by the biochemical analyses of oxidative stress parameters, functional analysis using electroretinography, and histology.

**Table 1:** Properties of compounds **4** and **8** *in vitro*.

Compound Properties	Compound 4	Compound 8
Cu (II) Binding Stoichiometry*	2.0 ∶ 1.0	2.0 ∶ 1.0
Fe (II) Binding Stoichiometry*	1.0 ∶ 2.0	2.0 ∶ 2.1
Zinc Binding Stoichiometry*	1.0 ∶ 2.1	2.0 ∶ 2.0
HLEC Viability#	72%	75%
RPE Viability#	75%	71%

*Stoichiometry (Compound ∶ Metal Ion) of complexes formed in solution as determined by Job Plots [23].

# Percent viable human lens epithelial cells (HLECs) and retinal pigmented epithelium (RPE) cells after 2 hour exposure to 1 mM Fenton reagents (1 mM H_2_O_2_ and Fe^2+^) [23].

## Materials and Methods

All reagents employed were of reagent grade. 4-(5-hydroxypyrimidin-2-yl)-N,N-dimethyl-3,5-dioxopiperazine-1-sulfonamide (**4**) and 4-(5-hydroxy-4,6-dimethoxypyrimidin-2-yl)-N,N-dimethyl-3,5-dioxopiperazine-1-sulfonamide (**8**) (99% pure by HPLC) were synthesized as previously described [23]. Antibodies used: anti-TRx (#2429; Cell Signaling Technology, Danvers, MA) 1∶1000 and anti-TRxR (#ab16840; Abcam, Inc., Cambridge, MA) 1∶2000. Chemiluminescent reagent and peroxide, biotinylated protein ladder, and pre-stained protein marker were obtained from Cell Signaling Technology (Danvers, MA). Polyacrylamide Ready Gels (4–15% Tris-HCl) and Trans-Blot® nitrocellulose membrane were obtained from Bio-Rad Laboratories (Hercules, CA). OxiSelect™ HNE-His Adduct and OxiSelect™ Nitrotyrosine ELISA Kits were purchased from Cell Biolabs, Inc. (San Diego, CA).

### Animal Care

All procedures were performed in strict accordance with the recommendations in the Guide for the Care and Use of Laboratory Animals of the National Institutes of Health and approved by the Institutional Animal Care and Use Committee of UNMC (Permit Number: 09-039-05).

### Oral Administration of Multi-functional Compounds

Four to five-week-old male Wistar rats (Charles River Laboratories, Wilmington, MA), divided into three groups of 24 animals each, were maintained under total darkness for the duration of the experiment except for twice daily checks under dim red light (5 lx). One group was fed standard rodent chow while the other two groups received standard rodent chow supplemented with 0.05% of either compound **4** or **8** for the duration of the experiment. Efficacy studies require the comparison of an endpoint, such as ONL thickness, when both compounds are administered at 50% of their effective dose (ED_50_), which is not currently known. Therefore, the concentration of supplementation in diet (0.05%) was based on reports of orally administered antioxidants that have been shown to reduce light-induced retinal damage [19], [20], [24], [25]. Rats received the compound of interest in the diet rather than by gavage to minimize the potential of effects due to half-life. Since rats tend to eat while active, the administered dose is obtained over time rather than a bolus dose. Rats were housed four to a cage and water was provided ad libitum. Animal weight and food intake was measured at 3–4 day intervals.

### Light-induced Retinal Damage

After two weeks of dark adaptation, 12 rats from each group were individually placed into acrylic, flat bottom rodent restraints (Plas-Labs, Inc, Lansing, MI) in a light box apparatus and exposed for three hours to 1000 lx of cool white fluorescent light (Lights of America, Los Angeles, California) (light-damaged rats, LD). The remaining 12 rats in each group were also placed into the light box apparatus for three hours, but not exposed to light (non-light-damaged rats, NLD). Oxidative stress markers were evaluated in 8 LD and 8 NLD rats of each group immediately after light exposure. Following euthanasia, the neural retinas were carefully dissected from the enucleated eye and frozen on dry ice. The neural retina from the left eye was evaluated for neural retinal levels of compounds while the right neural retina analyzed for oxidative stress. For functional and morphological assessment, the remaining 4 LD and 4 NLD rats in each group were returned to the dark environment after exposure and retinal function was assessed by ERG, 5 to 7 days later. Following ERG analysis, the rats were euthanized and the enucleated eyes were immediately processed for quantitative morphology.

### Oxidative Stress Determination

Retinas were homogenized in lysis buffer (50 mM Tris HCl, pH 7.5; 100 mM NaCl; 10 mM EDTA) for ELISA tests or in RIPA buffer (Cell Signaling Technology, Danvers, MA) containing Halt Protease and Phosphatase Inhibitor Cocktail (VWR International, West Chester, PA) for western blots. Total protein concentrations of the retinal homogenates was determined by the Bradford method [26]. Using the 4-HNE-His Adduct and Nitrotyrosine ELISA Kits, modified protein levels were measured according to manufacturer procedures except as follows. 4-HNE levels were determined in 100 µL aliquot of retinal homogenate (100 µg/mL) and nitrotyrosine-modified protein levels were evaluated in a 50 µL aliquot of retinal homogenate (200 µg/mL). Absorbance at 450 nm was read on a SpectraMax Plus^384^ spectrophotometer (Molecular Devices, Sunnyvale, CA). The level of HNE-protein adducts and nitrotyrosine-modified proteins were determined from constructed standard curves. All assays were performed in triplicate.

For western blots, protein samples were separated by SDS-PAGE and transferred onto a nitrocellulose membrane. The membrane was blocked with 5% dry milk in 0.1% TBST and incubated overnight at 4°C with primary antibody. The membranes were washed with TBS-T and incubated with a HRP-conjugated secondary antibody for 1 hour. The specific protein bands were visualized by chemiluminescence and exposure to x-ray film, as determined by molecular weight markers. Optimization of western blot and chemiluminescent conditions were performed using recombinant thioredoxin (#ab51064; Abcam) and thioredoxin reductase (#ab95910; Abcam) protein standards [unpublished data]. To ascertain the comparative expression and equal loading of these protein samples, the membrane was re-probed with antibody against GAPDH as an internal control. The visualized protein levels were quantified using the NIH ImageJ program (http://rsbweb.nih.gov/ij/).

### Electroretinography (ERGs)

Each rat was prepared for ERG analysis under dim red light illumination. Pupils were first dilated with 1% atropine sulfate followed by anesthesia with an i.p. injection of ketamine (100 mg/kg) and xylazine (20 mg/kg). A drop of 1% carboxymethylcellulose was placed on the corneal surface of each eye to ensure electrical contact and to maintain corneal integrity. This was followed by the placement of the ERG electrodes (LKC Technologies Inc., Gaithersburg, MD). A needle electrode, placed under the skin of the forehead, served as ground. Body temperature was maintained at 38±0.3°C using a Themipaq heating pad. ERG recordings were performed according to Nystuen [27] using the UTAS system (LKC Technologies Inc., Gaithersburg, MD). Briefly, all stimuli generated were presented in a Ganzfeld chamber/light emitter. Dark-adapted (scotopic) responses were recorded over a 4.0 log unit range of intensities. Light-adapted (photopic) responses were obtained with white flashes (0.3 log unit step) after 10 min of exposure to the background light to allow for complete light adaptation. Flicker ERG analysis was not performed. The amplitude of the scotopic a-wave was measured from baseline to the a-wave trough, while the amplitude of the scotopic b-wave was measured from the trough of the a-wave to the peak of the b-wave. The photopic b-wave amplitude was measured from baseline to the b-wave peak. Photoreceptor protection was determined by comparing the average maximal amplitudes of the light-damaged rats from each group to the non-light damaged rats of the same group.

### Tissue Preparation

After ERG analysis, all animals were euthanized by carbon dioxide asphyxiation and the dorsal ventral orientation of each eye was marked with a cautery tool. The eyes were then enucleated and fixed in a 3∶1 (v/v) methanol∶ acetic acid solution overnight. The fixed samples were then processed by the UNMC Tissue Sciences Facility (http://www.unmc.edu/tissuesciences/).

### Measurement of the Outer Nuclear Layer Thickness

Digital images of the H&E stained sections were obtained with a Zeiss AxioPlan microscope (Carl Zeiss MicroImaging, Inc., Thornwood, NY) using Axiovision software. The outer nuclear layer (ONL) thickness was measured at 400 µm intervals, starting at the optic nerve head and extending toward the superior and inferior ora serrata using Image-Pro Plus software (Media Cybernetics, Silver Spring, MD).

### Neural Retinal Levels of Compounds 4 and 8

Neural retinas were homogenized in a Pellet Pestle® (Kimble Chase, Vineland, NJ) with 0.5 mL of PBS containing 0.4 mM N,N-Dimethyl-4-(pyrimidin-2-yl)piperazine-1-sulfonamide as the internal standard. The homogenates were centrifuged at 10,000 rpm for 5 min at 4°C and the protein concentration in an aliquot of the supernatant was determined according to Bradford [26]. The remaining supernatant was then deproteinized with equal volumes of zinc sulfate and barium hydroxide [28]. Following centrifugation at 10,000 *g* for 30 minutes at 4°C, the supernatant was dried *in vacuo* and the residue was dissolved in chloroform, filtered, and dried *in vacuo*. The remaining organic residue containing the internal standard and extracted compounds **4** or **8** was dissolved in 250 µL of HPLC grade acetonitrile and separated by reverse phase HPLC (Luna 5 µm C18, 250×4; Phenomenex Inc., Torrance, CA). The samples were eluted with 50% aqueous methanol. The eluent was monitored by UV at 247 nm and by ESI-MS on a Thermo Finnigan LCQ (Thermo Fisher Scientific, Waltham, MA). Samples were quantified against standard curves of compounds **4** and **8**. All analyses were conducted in triplicate.

### Cell Culture

RGC-5 and 661wc ells were cultured in Dulbecco's Modified Eagle's Medium (DMEM) media containing 10% fetal bovine serum (FBS), 4 mM L-glutamine, 1.5 g/L NaHCO_3_, and 1% penicillin/streptomycin solution. The cells were plated at a density of 1×10^4^ cells into chamber slides or 96 well plates and grown until 70–80% confluent. For compound evaluation, FBS-free media (blank and control) or FBS-free media containing 1 mM of either compound **4**, **8** or Trolox, the water soluble vitamin E analog, were added to each individual well/chamber. After 1 hr, an additional aliquot of FBS-free media was added to each blank well/chamber, and a similar volume of FBS-free media containing concentrated H_2_O_2_ immediately followed by the addition of concentrated ammonium iron (II) sulfate hexahydrate was added to each well/chamber for a final concentration of 1 mM H_2_O_2_ and 1 mM Fe^2+^. For apoptosis detection, all media was removed after 4 h exposure and the cells were rinsed twice with PBS and air dried. The cells were then fixed with freshly prepared 4% paraformaldehyde in PBS, pH 7.4 for 1 h at room temperature. After washing with PBS, the cells were treated with ice cooled 0.1% Triton X-100 in 0.1% sodium citrate, again rinsed. Apoptosis was then detected by terminal deoxynucleotidyl transferase-mediated dUTP nick-end labeling (TUNEL) assay, using a fluorescein *in situ* cell death detection kit (Roche Applied Science, Germany). The stained cells were then analyzed under a fluorescence microscope and the apoptosis index was calculated as the percentage of TUNEL-positive nuclei present in photographs taken under 200× magnification in 5 to 10 fields in each well. For cell viability studies, conducted in 96-well plates, media was removed after 5 h exposure and the cells were rinsed twice with PBS. The cells were then processed using the CellTiter 96® AQueous One Solution Cell Proliferation Assay (MTS, Promega, Madison, WI) as previously described [23].

### Statistical Analyses

The calculations and statistical analyses (ANOVA) were conducted using OriginPro® software version 8.1 (OriginLab Corp., Northampton, MA) and ProStat ver. 5.01 (Pearl River, NY). Differences with a p<0.05 were defined as significant.

## Results

### Induction of Experimental Light Damage

Four to five-week-old male Wistar rats (Charles River Laboratories, Wilmington, MA) were divided into three groups of 24 animals each and dark adapted by maintaining them in total darkness for two weeks except for twice daily examination under dim red light (5 lx). All rats received standard rat chow; however, one group received standard chow containing 0.05% wt% compound **4** and a second group received standard chow containing 0.05% wt% of compound **8**. Feeding records indicate that rats in these groups ingested average doses of 81.7±13.2 and 83.3±9.9 mg/kg/day (Mean±SEM), of compounds **4** and **8**, respectively, and no significant difference in body weights between any group was observed. After two weeks of dark adaption, one half of the rats from each group were individually placed into acrylic rodent restraints and exposed to 1000 lx of cool white fluorescent light for three hours (light damaged rats, LD). The remaining rats in each group were also placed into the light box apparatus for three hours, but not exposed to light (non-light-damaged rats, NLD). One half of the LD and NLD rats in each group were immediately analyzed for oxidative damage. The remaining rats in each group were returned to the dim light environment for 5–7 days for subsequent retinal functional analysis.

### Effects of Compounds 4 and 8 on 4-HNE- and Peroxynitrite-mediated Protein Modifications in the Neural Retina

**Figure 1:**
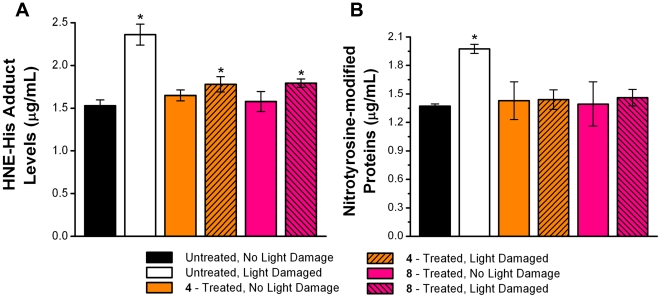
Comparison of 4-hydroxynonenal (HNE)-histidine adduct levels and nitrotyrosine adduct levels in the neural retina. **A**. HNE-histidine adduct levels, as determined by ELISA, in rats treated with/without compounds **4** and **8** suggests that HNE levels were reduced by treatment with multi-functional compounds. **B**. Similar conclusions were obtained with ELISA measurements of nitrotyrosine-modified proteins in rats treated with/without compounds **4** and **8**. Mean ± S.D.; n = 6. An asterisk (*) denotes a significant difference (p<0.05) when compared to the untreated, non light-damaged (NLD) group.

Immediately after light exposure, oxidative stress markers were evaluated in 8 LD and 8 NLD rats from each group. 4-Hydroxynonenal (4-HNE), generated by the non-enzymatic oxidation of n−6 polyunsaturated fatty acids, forms adducts with histidine (HIS) and can be used as a marker of oxidative stress [29], [30]. Analysis of these adducts by ELISA indicated that light exposure significantly increased the levels of 4-HNE-modified proteins in the neural retinas by 0.83 µg/mL (54%, P<0.05) compared to untreated, NLD rats (Figure 1A). However, neural retinal levels of 4-HNE did not significantly increase in light treated rats receiving either compound **4** or **8**.

Another oxidative marker is nitrotyrosine-modified proteins which results from tyrosine residues being attacked by peroxynitrite, a strong oxidant formed by the reaction of nitric oxide with superoxide anions that are generated by excessive light exposure [9], [31]. Analysis of nitrotyrosine modified protein levels in neural retinas by ELISA indicated that light exposure significantly increased nitrotyrosine levels by 44% (0.60 µg/mL, p<0.05) compared to untreated, NLD rats. This increase in nitrotyrosine levels was not observed in LD rats treated with either compound **4** or **8** (Figure 1B).

### Effects of Compound 4 and 8 on Thioredoxin and Thioredoxin Reductase Levels in the Retina

**Figure 2:**
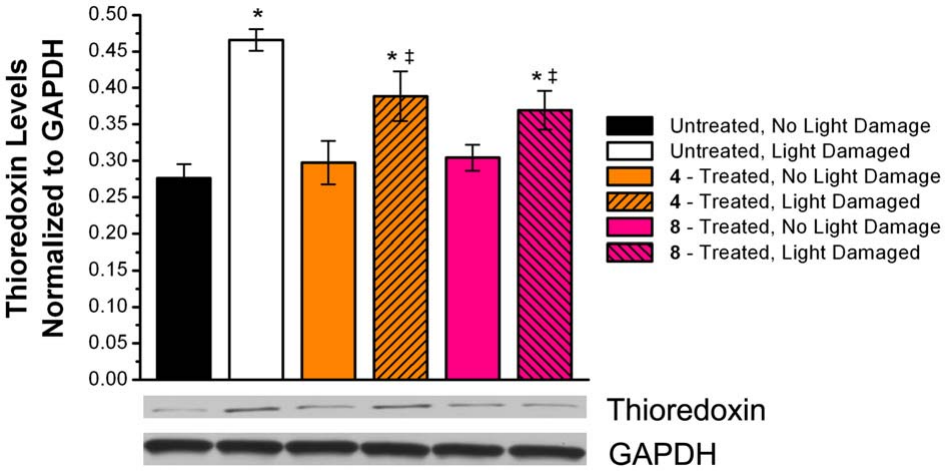
Expression of thioredoxin (TRx) in the neural retina. Top: Comparison of expression of TRx normalized to levels of GAPDH in rats treated with/without compounds **4** and **8** suggest that treatment with multi-functional compounds reduce induction of TRx. Retinal protein homogenate was subjected to Western blotting with anti-TRx and anti-GAPDH antibodies. Bottom: Lanes 1, control diet, non light-damaged; Lane 2, control diet, light-damaged; Lane 3, **4**-treated, non light-damaged; Lane 4, **4**-treated, light-damaged; Lane 5, **8**-treated, non light-damaged; Lane 6, **8**-treated, light-damaged. Lanes of interest were cropped from entire western blot scan prior to be auto leveled using Adobe Photoshop and analyzed by ImageJ software. Mean ± S.D.; n = 6. An asterisk (*) denotes a significant difference (p<0.05) when compared to the untreated, non light-damaged group. A double dagger (‡) denotes a significant difference (p<0.05) when compared to the untreated, light-damaged group.

**Figure 3:**
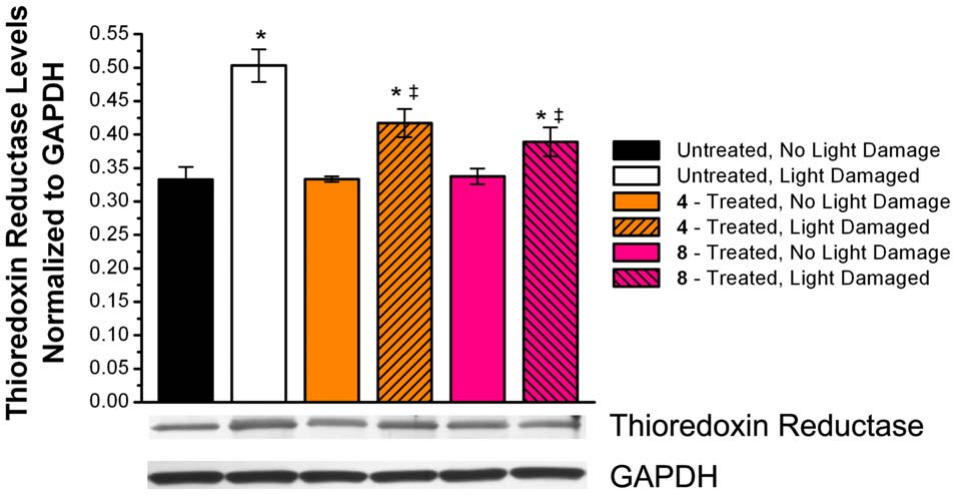
Expression of thioredoxin reductase (TRxR) in the neural retina. Top: Comparison of expression of TRxR normalized to levels of GAPDH in rats treated with/without compounds **4** and **8** suggest that treatment with multi-functional compounds reduce induction of TRxR. Retinal protein homogenate was subjected to Western blotting with anti-TRxR and anti-GAPDH antibodies. Bottom: Lanes 1, control diet, non light-damaged; Lane 2, control diet, light-damaged; Lane 3, **4**-treated, non light-damaged; Lane 4, **4**-treated, light-damaged; Lane 5, **8**-treated, non light-damaged; Lane 6, **8**-treated, light-damaged. Lanes of interest were cropped from entire western blot scan prior to be auto leveled using Adobe Photoshop and analyzed by ImageJ software. Mean ± S.D.; n = 6. An asterisk (*) denotes a significant difference (p<0.05) when compared to the untreated, non light-damaged group. A double dagger (‡) denotes a significant difference (p<0.05) when compared to the untreated, light-damaged group.

ROS and free radicals generated by retinal exposure to acute excessive light is mediated by superoxide dismutase, glutathione (GSH), and the thioredoxin (TRx)/thioredoxin reductase (TRxR) defense system [32], [33], [34]. As illustrated in Figures 2 and 3, intense light exposure induced the expression of both TRx and TRxR in the neural retinas of untreated rats. This induction of TRx reduced in the neural retinas of light exposed rats treated with either compound **4** or **8** (Figure 2). A similar reduction was also observed for the expression levels of TRxR (Figure 3).

### Functional Assessment of the Rat Retina by Electroretinography

**Figure 4:**
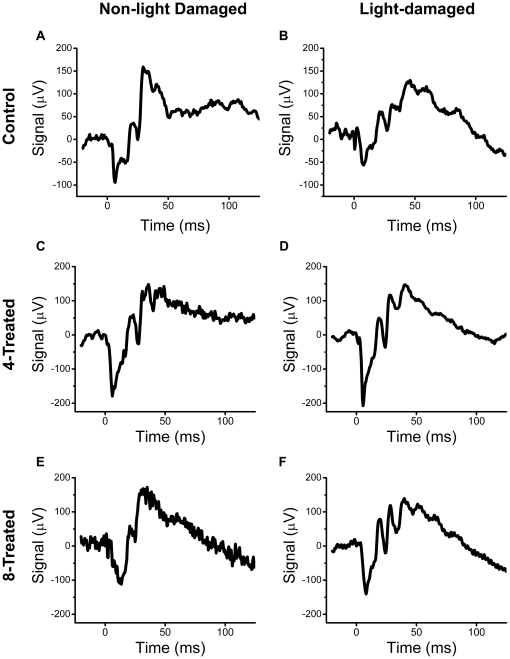
Dark-adapted (scotopic) ERG responses. Representative average maximal ERG scotopic response from untreated, control rats (A,B), 4-treated rats (C,D), and 8-treated rats (E,F). The a-wave was measured from baseline to trough, while the b-wave was measured from the trough of the a-wave to the peak of the wave.

**Figure 5:**
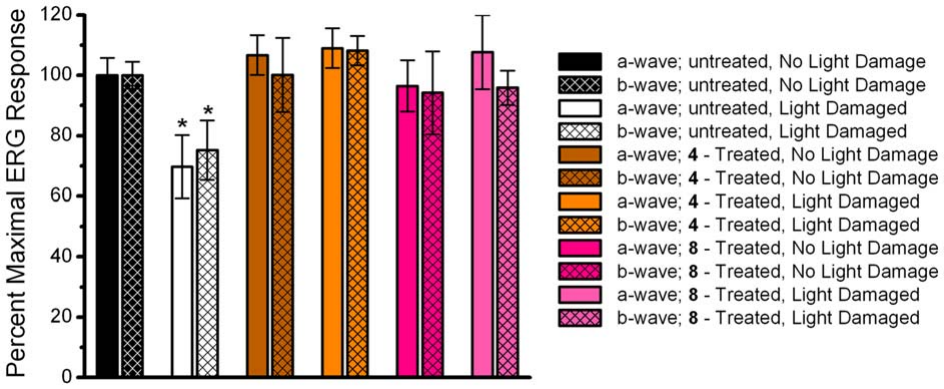
Percent dark-adapted ERG response. The mean (±S.E.M) of the maximal scotopic a-wave and b-wave amplitudes for non light-damaged and light-damaged eyes are shown as a percent of the maximal amplitude from untreated, non light-damaged rats (100% response). n = 6 rats. An asterisk (*) denotes a significant difference (p<0.05) when compared to the untreated, non light-damaged group.

**Figure 6:**
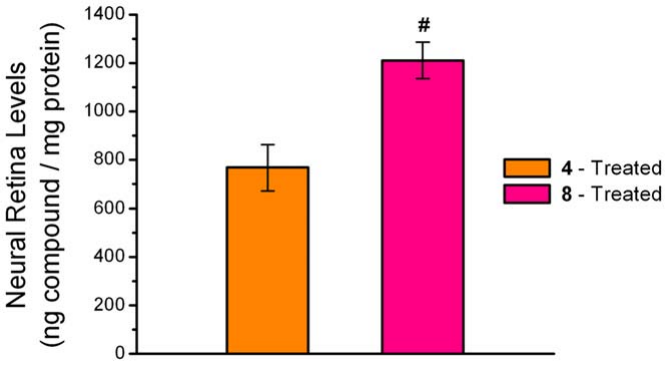
HPLC-MS analysis of neural retinal levels of 4 and 8. Analysis confirms accumulation of investigational compounds **4** and **8** in the neural retina following two week feeding of diet supplemented with 0.05% compounds **4** and **8**. Mean ± S.E.M.; n = 6. The pound (#) sign denotes a significant difference (p<0.05) when compared to the **4**-treated group.

**Figure 7:**
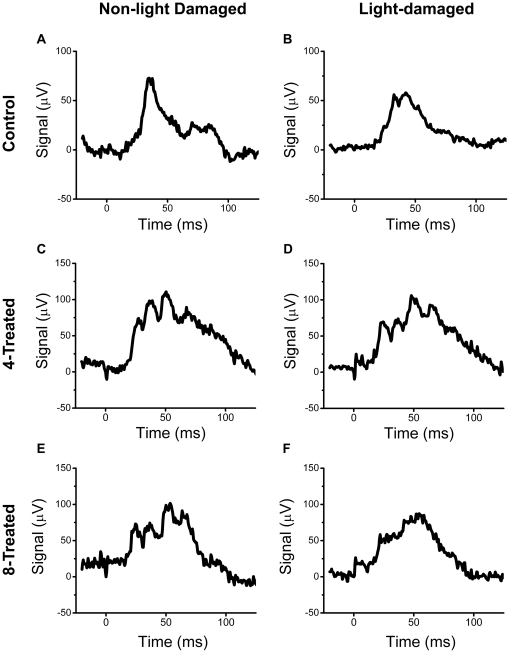
Light-saturated (photopic) ERG responses. Representative average maximal ERG photopic response from untreated, control rats (A,B), 4-treated rats (C,D), and 8-treated rats (E,F). The b-wave was measured from baseline to the peak of the wave.

**Figure 8:**
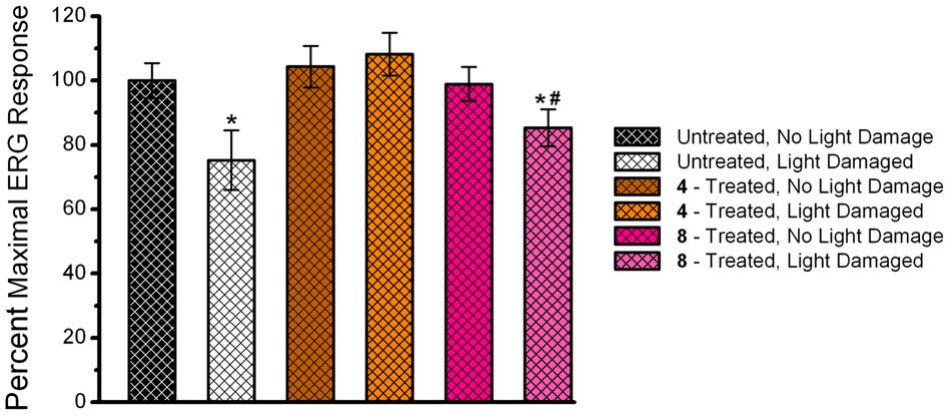
Percent light-saturated ERG response. The mean (±S.E.M) of the maximal photopic b-wave amplitude for non light-damaged and light-damaged eyes are shown as a percent of the maximal amplitude from untreated, non light-damaged rats (100% response). n = 6 rats. An asterisk (*) denotes a significant difference (p<0.05) when compared to the untreated, non light-damaged group. A pound sign (#) denotes a significant difference (p<0.05) when compared to the light-damaged, **4**-treated group.

In addition to generating ROS, intense light exposure enhances lipid peroxidation of the photoreceptor outer segments [11] which contributes to photoreceptor cell death [21], [35]. Following 5–7 days of dark recovery from intense light exposure, the retinal neural functions of the remaining rats in each group (and their corresponding non-light damaged controls) were evaluated by non-invasive electroretinography (ERG), a procedure that measures the function of photoreceptors (rods and cones), inner retinal cells (bipolar and amacrine cells), and ganglion cells through the measurement of electrical responses [36], [37], [38]. Representative waveforms of the maximal ERG scotopic response from non-light damaged (left column) and light damaged (right column) control (A,B), **4**-treated (C,D), and **8**-treated (E,F) rats are illustrated in Figure 4. Compared to untreated, NLD control rats, the average scotopic maximum a- and b-wave amplitudes were significantly (p<0.05) reduced by 30.4% and 24.8%, respectively, in the untreated LD rats (Figure 5). This reduction in the scotopic maximum a- and b-wave amplitudes was absent in LD rats treated with compounds **4** and **8**. Furthermore, the NLD rats treated with compounds **4** and **8** displayed similar scotopic ERG responses (Figure 5), suggesting that in addition to protecting the retina against light damage, these compounds demonstrated no toxic effects that damage to the retina at the levels present (Figure 6). Representative waveforms of the maximal ERG photopic response from non-light damaged (left column) and light damaged (right column) control (A,B), **4**-treated (C,D), and **8**-treated (E,F) rats are illustrated in Figure 7. In the photopic ERG b-wave response, untreated LD rats demonstrated a significant (24.7%, p>0.05) reduction in maximal amplitude compared to untreated, NLD rats (Figure 8). Compared to the untreated LD rats, the photopic b-wave response of LD rats treated with compound **8** was only slightly reduced, while the b-wave amplitude from rats treated with compound **4** was similar to that of untreated, NLD rats (Figure 8). The b-wave amplitudes of NLD rats treated with **4**-treated and **8**-treated were also similar to that of untreated NLD rats (Figure 8), confirming that compounds **4** and **8** do not appear to be toxic to the neural retina.

### Morphological Evaluation of Light-induced Retinal Damage by Quantitative Histology

**Figure 9:**
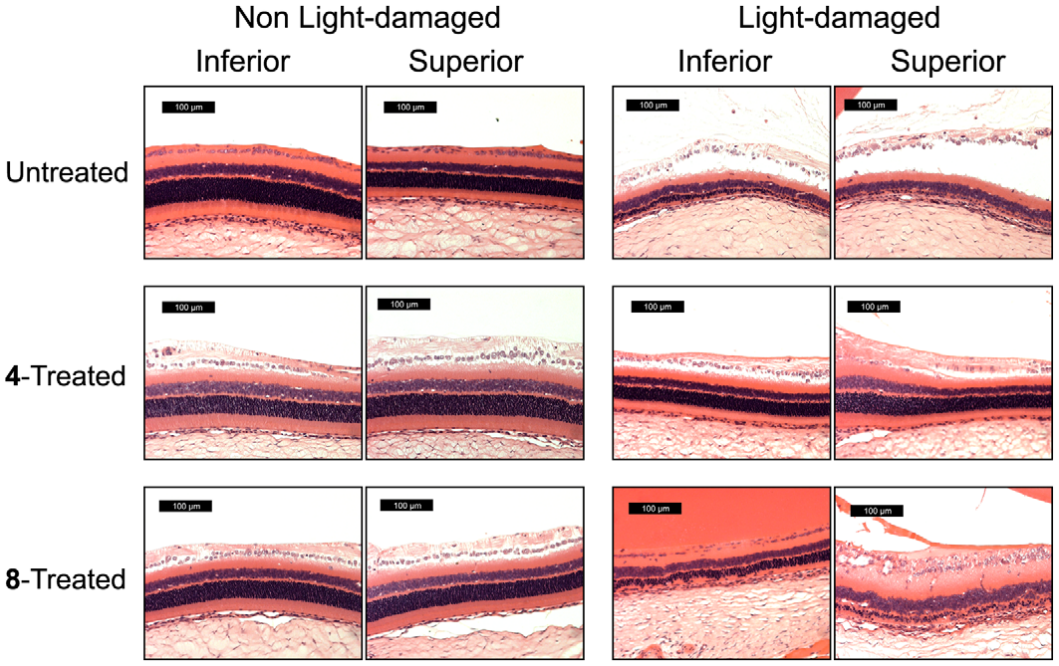
Histological sections of the ONL. Representative microscope images taken at 1 mm inferior and superior to the ONH of H&E stained sections through the optic nerve of retinas treated with/without compounds 4 and 8. Left: Non light-damaged (−) retinas treated with/without compounds 4 and 8. Right: ONL thickness of light-damaged (+) retinas treated with/without compounds 4 and 8. The white point (background) in each image was set using Adobe Photoshop prior to analysis of ONL thickness using Image-Pro Plus software.

The observation that rats treated with either compound 4 or 8 showed a decrease in oxidative stress, leading to little or no loss of retinal function was confirmed by histology. Intense light exposure results in photoreceptors thinning in the outer nuclear layer (ONL), with damage in the superior hemisphere more pronounced than the inferior hemisphere [6]. Retinal sections transversing the optic nerve head were prepared from rats in each group and the thickness of the ONL of the retina was measured from the optic nerve head to the inferior and superior ora serratas using image analysis software. Example high magnification images of the retinal sections taken one millimeter superior and one millimeter inferior to the optic nerve head for each group are illustrated in Figure 9.

**Figure 10:**
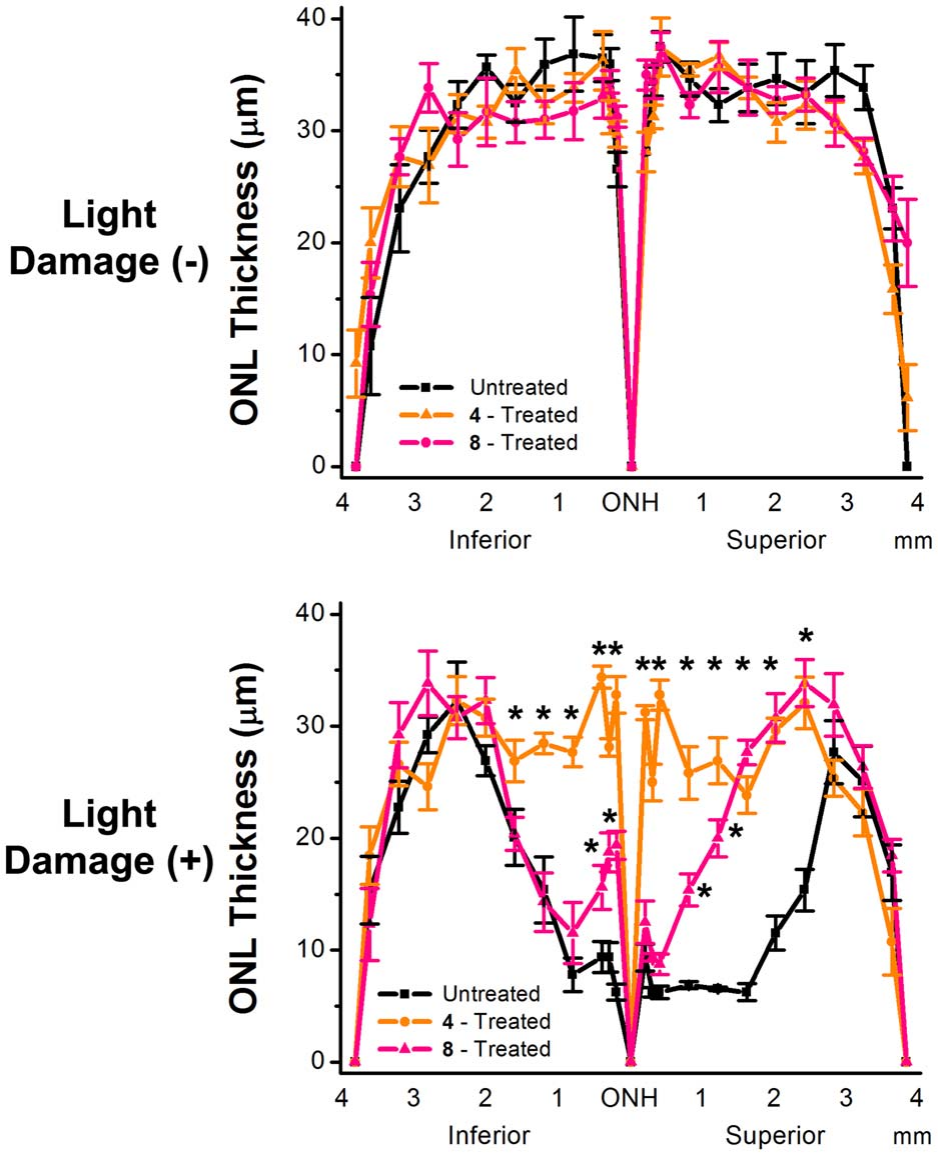
Retinal outer nuclear layer (ONL) thickness. The ONL thickness of the inferior and superior hemispheres was measured in µm at each defined distance from the optic nerve. Top: ONL thickness of non light-damaged (−) retinas treated with/without compounds **4** and **8**. Bottom: ONL thickness of light-damaged (+) retinas treated with/without compounds **4** and **8**. n = 3. Mean ± S.E.M. An asterisk (*) denotes a significant difference (p<0.05) when compared to the untreated, light-damaged group.

Histological evaluation of the ONL indicated a significant 40% (p<0.05) thinning of the photoreceptor layer in the inferior hemisphere and a 62% (p<0.05) thinning in the superior hemisphere (Figure 10) compared to NLD rats. This light induced thinning of the ONL was significantly (p<0.05) reduced in LD rats treated with compounds 4 and 8. Compared to untreated LD rats, the ONL was reduced 26% in the inferior and 35% in the superior hemisphere in rats treated with compound 8 (Figure 10). In the rats treated with compound 4, the photoreceptor layer was decreased 4% in the inferior hemisphere and 21% in the superior hemisphere (Figure 10). In the NLD rats no difference in ONL thickness was observed between the untreated and compound 4 or 8 treated rats confirming that these compounds are not phototoxic (Figure 10).

These histological results complement the ERG studies by confirming that oral administration of compounds 4 or 8 reduced oxidative stress generated by light damage and protected the structure and function of the retina. Evaluation of retinal levels of drug in select rats from each group revealed that compounds 4 and 8 achieved neural retina levels of 770±90 ng/mg protein and 1210±75 ng/mg, respectively (Mean±SEM) (Figure 6). While in vitro culture studies showed compounds 4 and 8 to be equipotent, the better protective effects of compound 4 combined with its lower retinal tissue levels suggests that compound 4 is more potent that compound 8 in vivo.

### Effects of Compound 4 and 8 on Cell Viability and TUNEL Staining *in vitro*

**Figure 11:**
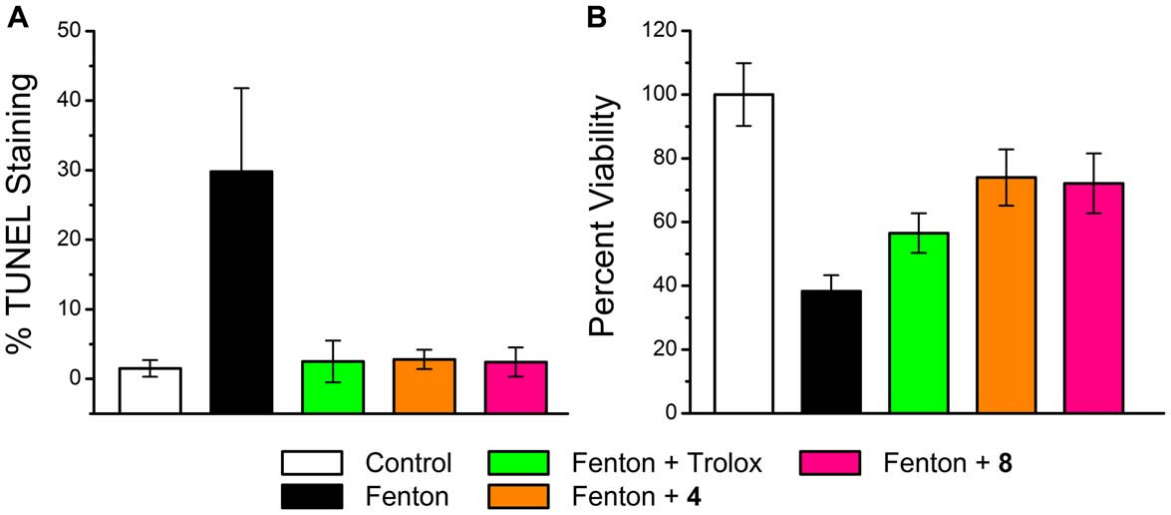
Protective effects of compounds 4, 8 and Trolox against Fenton-generated ROS. **A**. Protection against apoptosis detected by TUNEL staining of retinal ganglion RGC-5 cells cultured for 4 h in oxidative conditions generated by 1 mM H_2_O_2_ and 1 mM Fe(II). Mean ± SD, n = 4. **B**. Cell viability detected by the spectrophotometric MTS assay of 661w photoreceptor cells after 5 hr exposure to ROS generated by 1 mM H_2_0_2_ and 1 mM Fe(II). Mean ± SD, n = 6.

While we have previously demonstrated the efficacy of these multi-functional compounds in a human lens epithelial cell line, human retinal pigmented epithelial cell line and a human hippocampal astrocyte cell line [23], the present neuroprotective studies are focused on the photoreceptor layer. To confirm that these multifunctional compounds also protect retinal cells associated with this layer, *in vitro* cultured rat ganglion RGC-5 cells and murine 661w photoreceptor cells were cultured in the presence of 1 mM Fenton reagents (1 mM H_2_O_2_ and 1 mM Fe^2+^) with/without the water-soluble α-tocopherol analog Trolox, compound **4** or compound **8**. As summarized in Figure 11, multi-functional compounds **4** and **8** provided Trolox-equivalent protection against apoptosis, as measured by the TUNEL assay (Fig 11A), and Trolox-superior protection of the photoreceptor cells, as measured by the MTS cell viability assay (Fig. 11B).

## Discussion

Light-induced retinal degeneration in animals has been extensively used to evaluate neuroprotective candidates for the treatment of retinal degenerations such as AMD because light-damaged photoreceptors undergo oxidation-induced apoptosis similar to that observed in AMD [39], [40]. An important mechanism in light-induced retinal injury is hydroxyl radical-mediated lipid peroxidation that is propagated through Haber-Weiss and Fenton reactions catalyzed by iron or copper [10], [11], [41], [42]. Since these reactive oxygen species are short lived, this has been indirectly confirmed through the use of antioxidants [18], [19], [20], [21], [22] and chelating agents, such as desferrioxamine [17], both of which demonstrate some effectiveness in ameliorating retinal damage *in vivo*. The multi-functional antioxidants, 4-(5-hydroxypyrimidin-2-yl)-N,N-dimethyl-3,5-dioxopiperazine-1-sulfonamide (**4**) and 4-(5-hydroxy-4,6-dimethoxypyrimidin-2-yl)-N,N-dimethyl-3,5-dioxopiperazine-1-sulfonamide (**8**) used in the present study have the independent ability to both attenuate redox-active metals and scavenge free radicals. *In vitro* they reduced ROS-induced human RPE damage and selectively chelated iron or copper (Table 1). These compounds contain a newly described 2-amino-5-hydroxy-1,3-pyrimidine free radical scavenging system [43] and, to our knowledge, this is the first *in vivo* study that uses this unique radical scavenging system and the first *in vivo* study to demonstrate a protective effect of an orally active combination metal chelator and free radical scavenger on light-induced retinal degeneration. While it is well-established that light damage to the photoreceptor layer results from the generation of ROS and the subsequent release of free iron from the degenerating photoreceptors, the present study could not determine the relative contributions of free radical scavenging versus chelation demonstrated by the multi-functional compounds. To determine the detailed importance of these two factors, new experiments utilizing analogs with only chelating or free radical scavenging activity as described by Jin et al [23] will have to be conducted.

Unlike many metal binding compounds, these multi-functional antioxidants are orally bioavailable. This is advantageous because oral administration is associated with high patient compliance. In the absence of detailed pharmacokinetic data, rats in the present study were administered compounds **4** and **8** in standard rodent diet to minimize drug elimination effects. Since rats continuously eat when active, they were continuously receiving drug. Wistar rats were utilized in the present study because they are more sensitive to light-damage compared to Sprague Dawley rats [24]. The intensity and duration of light exposure was based on reports that a 3 hour exposure to 1000 lx of significantly reduces ERG response in dark-adapted rats without completely abolishing the photoreceptor layer [24].

Since the rats were preloaded with compounds **4** and **8** in the present study prior to light exposure to ensure that adequate compound was present in the retina, these studies are prevention rather than intervention studies. Moreover, the concomitant preloading of compounds **4** and **8** in the dark controls represent a quick assessment of toxicity since potential phototoxicity of these compounds would be reflected in the ERG analysis of the dark controls treated with / without compounds. To determine intervention efficacy, these studies will have to be repeated with the compounds administered at time points from the time of photodamage to select periods after the light insult.

The retina is sensitive to oxidative stress and the persistent oxidative stress generated during light exposure initiates modification of retinal proteins by HNE and peroxynitrite [11], [41]. Consistent with previously published results [30], protein modification by 4-HNE and peroxynitrite was significantly increased following 3 hour exposure to 1000 lx of light. The modification of retinal proteins immediately following oxidative insult was effectively inhibited by oral administration of compounds **4** and **8**. Since treatment with compounds **4** and **8** reduced the end products of lipid peroxidation, it can be deduced that both of these compounds reduced the oxidative burden in the retina immediately following light-damage. These *in vivo* results support our findings *in vitro* [23].

Thioredoxin (TRx) defends against cellular oxidative damage by scavenging oxidative stress generated by ROS [32]. It is actively induced in the mitochondria of RPE cells upon oxidative stress, including acute light-induced injury [33]. Similarly, thioredoxin reductase (TRxR), has been shown to be induced under conditions of persistent oxidative stress [44]. The TRx/TRxR defense system helps combat ROS and free radicals produced in the neural retina by the acute exposure to light [45]. In the present study, the light-induced oxidative stress induced retinal expression of both TRx and TRxR in LD rats. In LD rats pretreated with **4** and **8**, TRx induction was partially reduced, but not to basal levels (NLD). The expression of TRxR followed a similar trend. This data suggests that compounds **4** and **8** reduced the presence of oxidative stress in the neural retina during and immediately following acute exposure to light.

Rod outer segments in the photoreceptor layer of the retina are rich in polyunsaturated fatty acids, which are most vulnerable to lipid peroxidation [46]. Lipid hydroperoxide accumulates in rat rod outer segments when exposed to constant illumination [42]. Lipid peroxidation in the rod outer segments leads to disruptions of the surrounding plasma membranes and cellular material escaping into the subretinal space [47]. The outer segments of cones are more susceptible to damage than rod outer segments. Following light-induced damage, ultrastructural changes such as an increase in the degree of waviness of the discs, breaks in the disc stacking, and complete disorientation of the discs in the cone outer segments have been observed [47]. While light damage to the rods and cones in the photoreceptor layer cannot be detected by ophthalmoscope, even at a time when animals are functionally blind [48], early changes can be detected by electroretinography (ERG) [48], [49]. In untreated, LD animals, a reduction of the maximal amplitudes of the scotopic a- and b-wave and the photopic b-wave response was observed. ERG scotopic and photopic responses were normalized in light-damaged rats by pre-treatment with compound **4**. Pre-treatment with compound **8** was able to retain similar maximal scotopic a-wave and b-wave amplitude, but did not completely protect the maximal photopic b-wave amplitude. Differences in *in vivo* efficacy of compound **8** between reduction of oxidative stress markers and maintenance of ERG signal could be due in part to reduced protection of the photoreceptors from secondary damage during the 5 to 7 days post light damage. No difference in ERG response in NLD animals treated with/without compound **4** or **8** was observed, suggesting that these compounds are not neurotoxic to the retina. These ERG results were confirmed by histology. Significant thinning of the ONL occurred in untreated, LD animals, with the superior hemisphere displaying more pronounced damage than the inferior hemisphere. In similar rats treated with compounds **4** and **8**, the light-induced thinning of the inferior and superior hemispheres of the ONL was significantly reduced, with compound **4** protecting better than compound **8**. Even though levels of compound **4** in the neural retina were lower than that of compound **8**, thinning of the ONL was significantly less in **4**-treated animals. Interestingly, rats treated with compound **8** demonstrated good ERG responses (Figures 5 and 8) despite greater histological damage compared to that seen in rats treated with compound **4** (Figures 9 and 10). Light damage is generally more severe in the superior versus the inferior hemisphere, as confirmed in the present study. Since the ONL damage was less in rats treated with compound **8** compared to the untreated light damaged controls, we conclude that the minimum number of photoreceptors to give a good ERG response must have been retained by the **8**-treated rats and that these signals predominantly came from the inferior hemisphere.

Since compounds **4** and **8** demonstrated similar *in vitro* activity [23] and protection against acute oxidative insult, further studies are required to explain the *in vivo* efficacy observed. While levels of compound **8** were higher in the retina, this level was not toxic to the photoreceptors, as indicated by both **8**-treated, NLD ERG responses and histology. The morphological evaluation of retinal damage confirmed the ERG results that multi-functional compounds **4** and **8** demonstrate neuroprotection. The average thickness of the ONL across the entire retinal expanse in NLD rats treated with/without compounds **4** and **8** was 30–35 µm with no significant difference between groups. This average thickness is less than that reported for Wistar pups born under dim cyclic light (40–45 µm) [24], suggesting that normal light exposure of these very light-sensitive rats prior to the onset of this study had already reduced ONL thickness. While it would have been ideal to wean our own Wistar pups under dim cyclic light to avoid this difference, this was not feasible to perform at UNMC at the time of these studies. Nevertheless, these studies remain valid because light exposure to the untreated dark adapted rats resulted in significant oxidative, ERG and histological changes that were absent in the similar dark maintained controls.

For the biochemical analysis of oxidative stress and drug levels, neural retinas were obtained and frozen immediately following light exposure. The neural retina from each left eye was evaluated for drug levels in the neural retina while the right neural retina was analyzed for oxidative stress. Due to the potential for protein degradation, the retinas of these rats were not perfused with PBS to remove blood from the neural retina. Since this can result in high retinal drug levels due to the present of drug in the blood, additional studies on two week oral administration of compounds **4** and **8** have been conducted (data not shown). PBS perfusion of the animals under anesthesia resulted in a 20% reduction of neural retina drug levels obtained. This does not invalidate our conclusion that the observed biochemical, physiological and morphometric results are linked to the presence of compound in the neural retina.

In our previous in vitro study, multi-functional compounds 4 and 8 reduced ROS-induced damage to human RPE cells, a cell type instrumental in the development of age-related macular degeneration [23]. AMD is a complex disease whose progression is linked to ROS, primarily H_2_O_2_, and the age-related accumulation of redox active metals [50], [51]. This Fenton-catalyzed oxidative stress is believed to trigger biochemical cascades that lead to progressive degeneration [51], [52]. This hypothesis has been indirectly confirmed by the effectiveness of the metal chelator, deferiprone, to reduce iron overload-induced retinal damage in a transgenic mouse model [53] and desferrioxamine's ability to protect against light-induced retinal damage [17], presumably by chelating iron released from ferritin by light [7]. Therefore, it was hypothesized that multi-functional antioxidants 4 and 8 should be neuroprotective in a rat model of light-induced retinal damage. While these compounds have the independent ability to scavenge free radicals and chelate metals, the present study only demonstrates total antioxidant ability (metal chelation plus scavenging free radicals) of these multi-functional compounds to protect the photoreceptor layer against intense oxidative-induced apoptosis. To determine the relative importance of free radical scavenging versus metal attenuation, future studies using compounds possessing only free radical scavenging or metal chelation ability as described by Jin et al are required [23]. The present study suggests that multi-functional compounds may be effective candidates for preventive therapy of AMD. However, further investigation in other animal models of AMD is required.
